# Variability in HOMA-IR, Lipoprotein Profile and Selected Hormones in Young Active Men

**DOI:** 10.1155/2013/412764

**Published:** 2013-11-14

**Authors:** Anna Keska, Grazyna Lutoslawska, Anna Czajkowska, Joanna Tkaczyk, Krzysztof Mazurek

**Affiliations:** ^1^Department of Biochemistry and Biology, Józef Pilsudski University of Physical Education, P.O. Box 55, 01-968 Warsaw 45, Poland; ^2^Department of Physiology, Józef Pilsudski University of Physical Education, P.O. Box 55, 01-968 Warsaw 45, Poland; ^3^Department of Sports Medicine, Józef Pilsudski University of Physical Education, P.O. Box 55, 01-968 Warsaw 45, Poland

## Abstract

Resistance to insulin actions is contributing to many metabolic disturbances. Such factors as age, sex, nutrition, body fat, and physical activity determine body insulin resistance. Present study attempted to asses insulin resistance and its metabolic effects with respect to energy intake in young, lean, and active men. A total of 87 men aged 18–23 participated in the study. Plasma levels of glucose, insulin, lipoproteins, cortisol, and TSH were determined. Insulin resistance was expressed as Homeostasis Model Assessment for Insulin Resistance (HOMA-IR) and calculated using homeostatic model. The median value of HOMA-IR (1.344) was used to divide subjects into two groups. Men did not differ in anthropometric parameters, daily physical activity, and plasma TSH and cortisol levels. However, in men with higher HOMA-IR significantly lower daily energy intake was observed concomitantly with higher TG, TC, and HDL-C concentrations in plasma versus their counterparts with lower HOMA-IR. Exclusively in subjects with higher HOMA-IR significant and positive correlation was noted between HOMA-IR and TC and LDL-C. We concluded that despite a normal body weight and physical activity, a subset of young men displayed unfavorable changes in insulin sensitivity and lipid profile, probably due to insufficient energy intake.

## 1. Introduction

Several studies have demonstrated that insulin is involved in the regulation of peripheral carbohydrate, lipid, and protein metabolism as well in the central regulation of energy homeostasis [1, 2]. It suppresses hepatic glucose and triglyceride production, inhibits adipose tissue lipolysis, whole body, and muscle proteolysis, and stimulates glucose uptake in muscles [3].

In consequence, resistance to insulin (IR) is contributing to the development of many metabolic disturbances. It was shown that resistance to insulin-stimulated glucose uptake, especially in adipose tissue, liver, and muscle brings about type 2 diabetes, one of the most common chronic diseases [4, 5]. Insulin resistance also interferes with lipid metabolism leading to atherosclerosis, hypertension, and cardiovascular disease (CVD) [6].

It is well documented that insulin sensitivity varies due to many factors, including age, sex, nutrition, and physical activity [7]. Insulin sensitivity decreases with age due to increasing body fat stores, especially in the abdominal region [8].

Women appeared to be more sensitive to insulin action both in the liver and in muscles. Dissimilarities in insulin sensitivity between men and women are probably attributed to fundamental differences in total adiposity, muscle mass, and fat distribution (i.e., visceral versus subcutaneous fat) [9].

It is well documented that excessive energy supply and subsequent overweight and/or obesity adversely affect insulin action [10]. On the other hand, it has been demonstrated that also fasting decreases insulin sensitivity [11].

However, the underlying mechanisms inducing insulin resistance in both conditions have not been completely elucidated. Based on existing studies, it appears that they are different, because the IR changes in obesity are associated with the increased plasma free fatty acid (FFA) levels, increased inflammatory cytokines, and excessive reactive oxygen species (ROS) production, while in fasting-induced IR exclusively an increase in FFA was observed [12].

An important factor positively affecting insulin sensitivity is physical activity. Both physically active individuals and athletes are characterized by higher insulin sensitivity and glucose effectiveness than sedentary controls [13].

However, it should be stressed that regular physical activity offers health benefits if it is accompanied by an adequate diet [14]. When the energy supply is insufficient or excessive in relation to energy expenditure, a number of metabolic disturbances appear, including altered insulin and other glucostatic hormones (e.g., cortisol and TSH) actions [15].

Metabolic effects of low energy availability as a consequence of dietary energy restriction and high energy expenditure have been thoroughly studied in females, since they cause many metabolic disturbances such as depressed ovarian hormone secretion, decreased bone mineral density, distorted lipoprotein profile, and endothelial dysfunction [16].

Data concerning effects of caloric restriction in young, healthy active males are limited. Hinton and Beck [17] have found that collegiate male athletes also are at risk of inadequate energy consumption. According to Pasiakos et al. [18] inadequate energy intake in both sexes has downregulated metabolic processes due to depressed synthesis of intracellular signaling proteins. Moreover, Loucks et al. [19] have emphasized that health risk factors of energy restriction are not limited to female subjects. 

Moreover, most studies concerning disturbances in insulin action with respect to physical activity and/or dietary habits concern middle-aged and/or older individuals with obesity and type 2 diabetes [20, 21].

Thus, this study was undertaken to evaluate metabolic profile and daily caloric intake in young, healthy active men with respect to calculated index of insulin resistance (HOMA-IR). 

## 2. Materials and Methods

### 2.1. Subjects

A total of 87 physical education male students were enrolled in our study. Participants were recruited by advertisements in student dormitories and by word of mouth. They were healthy, nonsmokers, and not taking medication on a regular basis. All the participants signed informed consent and the study has been approved by local Ethics Commission.

### 2.2. Anthropometric Methods

Body composition was estimated using the bioelectrical impedance method and BC 418 MA equipment (Tanita Co., Japan). Inter- and intra-assay coefficients of variation for body fat measurements did not exceed 5%.

### 2.3. Energy Intake

Participants were instructed to maintain their usual diet. Dietary intake was assessed for 4 days (two weekdays and weekend). Subjects provided 24-hour food records, which were next evaluated by an experienced interviewer using the Album of Photographs of Food Products and Meals. Nutritional data were analyzed using computer program Dieta 5.0. purchased in the National Food and Nutrition Institute in Warsaw [22]. Energy intake (EI) of each person was assessed individually, in relation to standards taking into account the body mass and physical activity.

### 2.4. Energy Expenditure

Activity energy expenditure (AEE) was evaluated using the Seven-Day Physical Activity Recall (SDPAR) questionnaire [23].

### 2.5. Biochemical Analyses

Fasting venous blood samples were collected into lithium heparin tubes using disposable syringes and needles under aseptic conditions. Blood was drawn between 7:30 and 9:00 a.m., centrifuged (15 min, 4000 rpm, 4°C), and stored at −70°C until analysis. Blood glucose levels were determined by the oxidase method. Plasma triglycerides (TG), total cholesterol (TC), and high-density lipoprotein cholesterol (HDL-C) were measured with colorimetric methods. All determinations were performed using Randox commercial kits (Randox Laboratories, UK). Inter- and intra-assay coefficients of variation for glucose, TG, TC, and HDL-C determination did not exceed 5%. Low-density lipoprotein cholesterol (LDL-C) was calculated using Friedewald formula [24].

Plasma levels of insulin, cortisol, and TSH were determined by standard radioimmunoassay methods using BioSource commercial kits (Belgium). Inter- and intra-assay coefficients of variation for hormones did not exceed 7%.

Insulin resistance was calculated by homeostasis assessment model (HOMA-IR) and calculated from fasting insulin and glucose concentration according to the formula: insulin (*μ*IU/mL) × glucose (mmol/L)/22.5 [25]. The median value of HOMA-IR (1.344) was used to divide study population into Group A (above the median, *n* = 43) and Group B (below the median, *n* = 44).

### 2.6. Statistical Analyses

Data distribution was assessed by the Shapiro-Wilk test. Data are expressed as means ± SD and the level of significance for all statistical tests was set at *P* < 0.05. Student's *t*-test or the Mann-Whitney test was used for comparison between groups, for normally and not normally distributed variables, respectively. Pearson product moment and Spearman rank correlation coefficients were calculated to evaluate the associations between variables. Data analyses were performed using Statistica v.9 (StatSoft, USA).

## 3. Results

The anthropometric, dietary, and metabolic characteristics of all subjects are listed in Tables 1 and 2. 

Tables 3 and 4 present data of subjects divided into Group A and Group B. As shown in Table 3 men from Group A did not differ significantly from men in Group B in body fat content. Furthermore, daily physical activity of participants expressed in kcal/day was similar. 

However, in men with higher HOMA-IR values significantly lower daily energy intake was observed in comparison to their counterparts with lower HOMA-IR (by 13.6%, *P* < 0.01). There were no differences in the percentage of energy from proteins, fats, and carbohydrates between groups.

In Group A plasma glucose and insulin levels were higher than in Group B (by 11.8% (*P* < 0.001) and 39.3% (*P* < 0.001) for glucose and insulin, resp.) (Table 4). Likewise, plasma TG, TC, and HDL-C concentrations were significantly higher in men with HOMA-IR higher the median (by 22.2%, *P* < 0.01, by 6.4%, *P* < 0.05, and 13.3%, *P* < 0.05, resp.). However, plasma cortisol and TSH levels were similar in both groups.

Exclusively in men with higher HOMA-IR (Group A) a significant and positive correlation was noted between HOMA-IR and TC, LDL-C (Table 5). 

## 4. Discussion

This study provided two important findings. First, we observed highly differentiated insulin resistance (estimated by HOMA-IR) in young lean men with similar body fat and activity level. Secondly, participants with higher HOMA-IR values were characterized by lower daily energy intake and less favorable lipoprotein profile.

However, from our data it is difficult to evaluate insulin resistance in participants of the study due to divergent results concerning HOMA-IR cutoffs. Conus et al. [26] have classified young women as insulin resistant based on a cut point of HOMA > 1.69, but Capasso et al. [27] have postulated HOMA-IR ≥ 2.5 as the cutoff value to define insulin resistance. 

On the other hand, mean HOMA-IR values in our participants (1.537) were higher than reported by others in young males (1.400–1.457), but close to that found in healthy middle-aged men (1.600) [28–30]. Thus, it could not be excluded that at least some of our participants were insulin resistant.

One of the important predictors of insulin sensitivity is caloric intake. Our results are in agreement with animal studies which have indicated elevated IR during caloric restriction [31]. 

The mechanism of this is not fully elucidated. However, it has been suggested that insulin resistance in response to energy deficiency is a survival strategy, which leads to increased fat oxidation in skeletal muscle and spares glucose for utilization by the brain [32, 33].

Additionally, it has been demonstrated that energy shortage brings about stimulation of catecholamine secretion adversely affecting insulin sensitivity [34, 35]. 

However, it should be noted that in our study difference in caloric intake between groups characterized by markedly different HOMA-IR was significant but relatively small. On the other hand, taking into account activity energy expenditure and subjects body mass, recommended caloric intake has to be closed to 3000 kcal/day [36]. Thus, subjects with lower HOMA-IR were close to recommended, but subjects with higher HOMA-IR were by 20% lower than recommended intake. 

Previous studies have postulated that reduced insulin sensitivity is associated with higher fat mass, lower fat free mass, and greater central fat accumulation [37]. In our study no significant differences in total body fat were found between men who varied in HOMA-IR values. However, it could not be excluded that our participants differed in visceral fat markedly affecting insulin sensitivity [38]. 

It was found that insulin resistance is associated with increased cardiovascular risk [39]. Many studies have shown that impaired insulin action is accompanied by atherogenic lipid profile, that is, elevated plasma levels of TG and LDL-C and decreased HDL-C [40]. 

In the current study young, active men with higher HOMA-IR (Group A) showed significantly higher fasting triglycerides and total cholesterol in comparison to Group B, which should be regarded as an unfavorable change in terms of health. 

Numerous data have demonstrated that circulating cortisol and TSH affect energy balance, lipoprotein profile, and insulin sensitivity [41, 42]. However, in our subjects plasma levels of both hormones did not differ irrespectively of caloric intake and HOMA-IR values. 

This possibly suggests that insufficient energy intake first of all leads to an increase in circulating insulin and HOMA-IR values. It seems that these changes precede that in body fat content, cortisol, and TSH levels due to caloric restriction [43]. Moreover, it could not be excluded that insulin sensitivity is a very subtle indicator of energy balance in physically active young men.

In conclusion, we found that despite a normal body weight and increased physical activity, a subset of young men display unfavorable changes in insulin sensitivity and lipid profile, probably due to insufficient energy intake. 

However, this study has several limitations and to confirm our results determination of basal metabolic rate and more precise determination of insulin sensitivity in young, active men are necessary. 

## Figures and Tables

**Table 1:** Characteristics of the 87 subjects.

Variable	Mean ± SD
Age (yr)	19.8 ± 0.8
Body mass (kg)	76.6 ± 8.8
Body height (cm)	180.6 ± 6.0
Fat (%)	12.6 ± 4.4
Fat (kg)	9.8 ± 4.2
AEE (kcal/day)	776.4 ± 353.5
EI (kcal/day)	2771.5 ± 690.9
Protein (g)	98.0 ± 27.6
Protein (% E)	14.5 ± 2.6
Fat (g)	114.7 ± 34.8
Fat (% E)	36.5 ± 5.8
Carbohydrate (g)	355.4 ± 100.7
Carbohydrate (% E)	49.5 ± 8.2

AEE: activity energy expenditure; EI: energy intake.

**Table 2:** Biochemical variables of the 87 subjects.

Variable	Mean ± SD	Reference values
Glucose (mmol/L)	4.8 ± 0.6	3.89–5.83
Insulin (*µ*IU/mL)	7.1 ± 2.7	1.0–25.0
TG (mmol/L)	0.81 ± 0.4	0.4–1.53
TC (mmol/L)	4.52 ± 0.7	<5.2
HDL-C (mmol/L)	1.43 ± 0.4	0.89–2.5
LDL-C (mmol/L)	2.68 ± 0.8	<3.5
Cortisol (nmol/L)	374.9 ± 148.0	131–642 (8–10 AM)
TSH (*µ*IU/mL)	2.1 ± 1.0	0.2–4.1
HOMA-IR	1.537 ± 0.736	—

**Table 3:** Characteristics of the subjects categorized by HOMA-IR median value (means ± SD).

Variable	Group A (*n* = 43) > median HOMA-IR	Group B (*n* = 44) ≤ median HOMA-IR
Age (yr)	19.7 ± 0.8	19.9 ± 0.8
Body mass (kg)	76.4 ± 8.4	76.4 ± 9.2
Body height (cm)	180.7 ± 6.0	180.5 ± 6.1
Fat (%)	13.3 ± 4.6	11.9 ± 4.2
Fat (kg)	10.4 ± 4.3	9.3 ± 4.1
AEE (kcal/day)	751.1 ± 362.9	801.1 ± 346.6
EI (kcal/day)	2569.4 ± 607.8**	2973.5 ± 716.9
Protein (g)	92.0 ± 22.3	104.0 ± 31.2
Protein (%)	14.7 ± 2.4	14.4 ± 2.8
Fat (g)	106.9 ± 31.4*	122.6 ± 36.6
Fat (%)	36.5 ± 4.5	36.5 ± 6.9
Carbohydrate (g)	327.1 ± 81.2**	383.6 ± 111.0
Carbohydrate (%)	48.8 ± 5.2	50.3 ± 10.4

AEE: activity energy expenditure; EI: energy intake; **P < 0.01, *P < 0.05 significantly different versus Group B.

**Table 4:** Biochemical variables in subjects differed by HOMA-IR (means ± SD).

Variable	Group A (*n* = 43) > median HOMA-IR	Group B (*n* = 44) ≤ median HOMA-IR
Glucose (mmol/L)	5.1 ± 0.5***	4.5 ± 0.4
Insulin (*µ*IU/mL)	8.9 ± 2.8***	5.4 ± 0.8
HOMA-IR	2.020 ± 0.781***	1.065 ± 0.163
TG (mmol/L)	0.9 ± 0.4**	0.7 ± 0.3
TC (mmol/L)	4.7 ± 0.8*	4.4 ± 0.6
HDL-C (mmol/L)	1.5 ± 0.4*	1.3 ± 0.3
LDL-C (mmol/L)	2.7 ± 0.9	2.6 ± 0.6
Cortisol (nmol/L)	391.1 ± 142.4	359.2 ± 153.2
TSH (*µ*IU/mL)	2.1 ± 1.0	2.0 ± 1.0

***P < 0.001; **P < 0.01; *P < 0.05 significantly different versus Group B.

**Table 5:** Correlation coefficients between HOMA-IR and biochemical variables in participants.

Variable	Group A (*n* = 43) > median HOMA-IR	Group B (*n* = 44) ≤ median HOMA-IR
TG (mmol/L)	0.12	0.08
TC (mmol/L)	0.42*	−0.03
HDL-C (mmol/L)	−0.17	0.02
LDL-C (mmol/L)	0.46*	−0.18
Cortisol (nmol/L)	0.10	−0.17
TSH (*µ*IU/mL)	−0.21	0.22

*P < 0.05.
